# Prognostic Value of Molecular Aberrations in Low- or Intermediate-Risk Neuroblastomas: A Systematic Review

**DOI:** 10.3390/cancers17010013

**Published:** 2024-12-24

**Authors:** Rixt S. Bruinsma, Caroline W. M. Lekkerkerker, Marta Fiocco, Miranda P. Dierselhuis, Karin P. S. Langenberg, Godelieve A. M. Tytgat, Max M. van Noesel, Marc H. W. A. Wijnen, Alida F. W. van der Steeg, Ronald R. de Krijger

**Affiliations:** 1 Princess Máxima Center for Pediatric Oncology, 3584 CS Utrecht, The Netherlands; 2 Department of Medical Statistics and Bioinformatics, Leiden University Medical Center, 2333 ZA Leiden, The Netherlands; 3 Mathematical Institute, Leiden University, 2333 CC Leiden, The Netherlands; 4 Division Imaging & Cancer, University Medical Center Utrecht, 3584 CX Utrecht, The Netherlands; 5 Department of Pathology, University Medical Center Utrecht, 3584 CX Utrecht, The Netherlands

**Keywords:** neuroblastoma, low-risk, intermediate-risk, non-high-risk, genetics, SCA, segmental chromosomal aberrations

## Abstract

Background: The 5-year prognosis of non-high-risk neuroblastomas is generally good (>90%). However, a proportion of patients show progression and succumb to their disease. We aimed to identify molecular aberrations (not incorporated in the current risk stratification) associated with overall survival (OS) and/or event-free survival (EFS) in patients diagnosed with non-high-risk neuroblastoma. Methods: We conducted a systematic search in PubMed, Embase, Cochrane and Google Scholar. Two reviewers independently and blindly screened titles/abstracts, references of protocols/reviews and full texts. Risk of bias was assessed using a customized Quality in Prognostic Studies tool. Applicability was assessed using a tool designed by the researchers. GRADE criteria were used to determine quality of evidence. Results: Sixteen studies (4718 patients) were included. A segmental chromosomal aberration (SCA) profile was associated with lower survival. 1p loss of heterozygosity (LOH) and 17q gain were associated with lower OS and EFS. 1p deletion and 2p gain were associated with lower OS, but this was not the same for EFS. 3p deletion was not associated with worse outcome. Quality of evidence was downgraded because of imprecision and publication bias and upgraded because of moderate/large effect, resulting in a moderate quality of evidence. Conclusion: The association of 1p LOH, 1p deletion, 2p gain and 17q gain with OS and EFS suggests that these SCAs may be added to the risk stratification to identify non-high-risk neuroblastomas with worse prognosis.

## Simple Summary

Neuroblastoma is the most common, non-central nervous system, solid tumor in children. The prognosis for non-high-risk neuroblastomas is generally good, but some patients still die because of their disease. We aim to identify DNA changes of the tumor that are associated with patient outcome. We conducted a systematic search in PubMed, Embase, Cochrane and Google Scholar and identified 16 articles related to this topic. The overall quality of evidence was moderate. DNA changes in chromosome arm 1p, 2p and 17q seem to be related to poor prognosis. Adding these DNA changes to the current risk stratification may help to identify non-high-risk neuroblastomas with worse prognosis and consequently adjusting their therapeutic approach.

## 1. Introduction

Neuroblastoma is a pediatric neural-crest-derived developmental tumor of the adrenal medulla and sympathetic side chains. It is known for its clinical and molecular heterogeneity with variable outcome [1,2]. The current standard treatment strategies subdivide patients into low- and intermediate-risk vs. high-risk disease [1]. Especially the low- and intermediate-risk patients are clinically very heterogeneous. This group contains children with tumors that can be treated by observation only, but also children with more aggressive disease, which requires full chemotherapeutic and local treatment [2].

In 1971, Audrey Evans developed the first International Neuroblastoma Staging System (INSS) based on clinical aspects such as age, surgical results, nodal status and tumor size. Since then, several other clinical and biological markers have been incorporated in the risk stratification of neuroblastomas [2]. Currently, the International Neuroblastoma Risk Group (INRG) classification system includes the following factors: disease stage, age at diagnosis, histological subgroup, tumor cell ploidy, *MYCN* status and 11q aberrations [1,3]. Based on this classification system, every neuroblastoma is classified into one of three categories: low, intermediate and high risk. The non-high-risk group (low-risk or intermediate-risk) includes patients with non-MYCN-amplified neuroblastoma stage L1, L2 (≤18 months or >18 months at diagnosis), MS and stage M ≤18 months at diagnosis [2]. On average the 5-year event-free survival (EFS) for patients with a non-high-risk neuroblastoma is 80–90% and 5-year overall survival (OS) is over 95% [4,5,6,7,8,9]. Treatment of non-high-risk patients varies from ‘watchful waiting’ to surgery, with or without (neo-)adjuvant chemotherapy [2]. This emphasizes the need for a more accurate risk stratification. MYCN has been recognized as prognostically unfavorable and is included in all stratifications. However, other aberrations have not been used systematically. The INRG has recognized 11q aberrations as factor for stratification [3], the German Gesellschaft für pädiatrische Onkologie und Hämatologie (GPOH) study group uses 1p loss of heterozygosity (LOH) [10], and the Children’s Oncology Group (COG) includes 1p and 11q LOH, 3p and 4p deletion, and 1q, 2p and 17q gain [11]. Over the last decades, several new recurrent genetic aberrations in neuroblastoma have been unraveled for which the prognostic value, especially in the non-high-risk group, is unclear [12,13,14,15]. This led us to perform a systematic review to address the role of molecular aberrations present in non-high-risk neuroblastomas.

More specifically, we aim to answer the following research question: which molecular aberrations (not incorporated in the current INRG risk stratification) are associated with OS and EFS in children (<18 years) initially diagnosed with a non-high-risk neuroblastoma?

## 2. Materials and Methods

### 2.1. Search Strategy and Selection Process

The research question was phrased according to the PICOTS framework [16] (Appendix A). A systematic search strategy was created for the databases PubMed, Embase, Google Scholar and Cochrane (Appendix B). We executed the search in September 2023 after consulting with a medical librarian. Following duplicate removal using Rayyan software [17], two reviewers (R.S.B. and C.W.M.L.) independently and blindly screened titles and abstracts. Inclusion criteria were studies investigating pediatric patients (<18 years old) initially diagnosed with a low-risk or intermediate-risk neuroblastoma, comparable to the definitions of the INRG classification system, that investigated molecular aberrations and their prognostic value. Studies that included only high-risk patients or in which they did not distinguish high-risk from non-high-risk neuroblastomas were excluded. However, studies that did not distinguish high-risk from non-high-risk, but did distinguish between *MYCN*-amplified and *MYCN*-non-amplified tumors were included. Applicability scores (see Section 2.2) of these studies were lower since patients > 18 months with stage 4 (or stage M) disease classify as high risk regardless of their MYCN status [3]. Molecular aberrations were defined as numerical chromosomal aberration (NCA), segmental chromosomal aberration (SCA), gene mutation or gene amplification. Studies investigating genomic profiles (SCA of NCA profile) were also included, but again applicability scores were lower (see Section 2.2). Studies that solely investigated molecular aberrations already incorporated in the INRG risk stratification (*MYCN* amplification and 11q aberrations) were excluded. Studies that classified patients using the revised COG classification system, in which the presence of an SCA’s potentially classifies a patient as high risk [11], were also excluded. Prognostic value had to be expressed in overall survival (OS), event-free survival (EFS) or progression-free survival (PFS). Furthermore, studies had to be written in English, German or Dutch. Other exclusion criteria were reviews and protocols (though they were used for snowballing purposes), animal studies, conference abstracts, non-peer-reviewed articles or articles of which no full text was available.

After title/abstract screening, disagreements between the reviewers were discussed to reach consensus. Furthermore, references of all excluded reviews and protocols identified through database search were screened in the same manner. If consensus could not be reached, a third reviewer (R.R.d.K.) was asked to resolve the discrepancy. Full-text screening was performed using the same method. Finally, articles written by the same author have been screened for overlapping patient groups. When two (or more) articles investigated the same prognostic factor(s) and there appeared to be an overlap in patients, only the article that included most patients was included.

This systematic review is in compliance with the PRISMA 2020 guidelines [18] (Supplementary Materials). The review was not registered and no protocol was prepared beforehand.

### 2.2. Applicability

An applicability tool was designed by the researchers specifically for this systematic review to assess applicability of all eligible studies (Appendix C). The applicability of each study was assessed based on the representativeness of their domain, determinant and outcome. Each criterion was scored ‘high’, ‘moderate’ or ‘low’. Assessment of applicability was performed by two reviewers (R.S.B. and C.W.M.L.) independently and blindly. Discrepancies were discussed to reach consensus.

### 2.3. Risk of Bias

The Risk of Bias (RoB) was assessed using a specified Quality in Prognosis Studies (QUIPS) tool. This tool examines the RoB across six domains: study participation, study attrition, prognostic factor measurement, outcome measurement, study confounding, and statistical analysis and reporting [19]. Judgement was made by two reviewers (R.S.B. and C.W.M.L.) independently and blindly. Discrepancies were discussed to reach consensus. Each domain was rated ‘low’, ‘moderate’, or ‘high’. Specifications can be found in Appendix D.

### 2.4. Data Extraction

We extracted the following data from the included articles: study design, setting (center and country), study population, number of patients included, molecular aberrations investigated, outcome variables and follow-up length. Furthermore, the OS and EFS/PFS with corresponding standard error (S.E.) or 95% confidence interval (CI) and *p*-value were reported. Unadjusted and adjusted prognostic factors were collected if adequately reported in the studies. For both hazard ratio (HR) and risk ratio (RR), the 95% CI or S.E. and the *p*-value were reported. A *p*-value of <0.05 was considered statistically significant in all included articles.

Data collection was independently and blindly performed by two reviewers (R.S.B. and C.W.M.L.).

### 2.5. Quality of Evidence

The Grading Recommendations Assessment, Development and Evaluation (GRADE) criteria were used to assess quality of evidence. Depending on these criteria, the quality of evidence was rated high, moderate, low, or very low. Observational cohort studies provide high confidence in prognostic studies. Therefore, a body of observational cohort studies starts out as high-quality evidence in a systematic review. There are five factors that may downgrade quality of evidence: RoB, inconsistency, imprecision, indirectness and publication bias. Three factors may upgrade quality of evidence: large effect, dose–response gradient, and plausible confounders decreas an apparent treatment effect [20]. The latter two are not applicable to this prognostic study [20,21]. Since multiple factors contribute to the prognosis of a health condition, both a moderate or large effect are reason to upgrade the quality of evidence in prognostic studies [21].

## 3. Results

### 3.1. Study Selection

**Figure 1:**
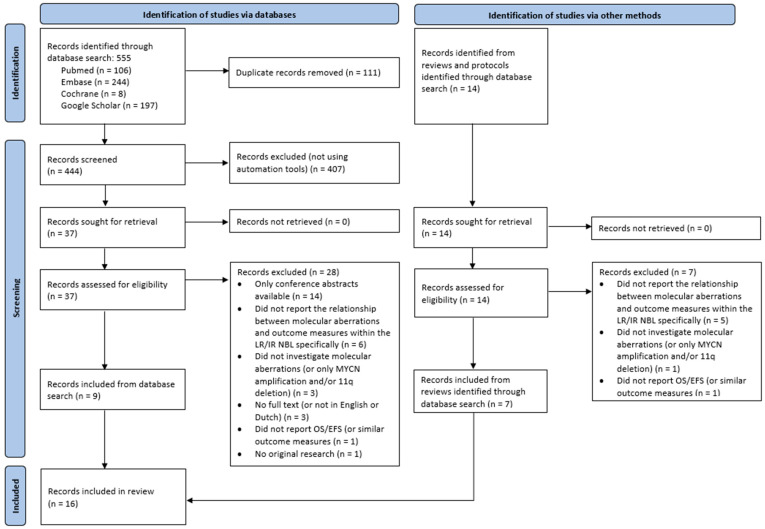
PRISMA 2020 flow diagram.

After duplicate removal, our systematic search yielded a total of 444 records. After title/abstract screening, 407 were excluded. The full text of the 37 remaining articles was screened and another 28 were excluded, thereby including nine records. Furthermore, references of reviews and protocols identified through database search were screened, which yielded another seven articles. An overview of the selection process can be found in Figure 1. Sixteen studies were considered eligible for inclusion, all of which were retrospective cohort studies [3,22,23,24,25,26,27,28,29,30,31,32,33,34,35,36]. Further details of the studies are described in Table 1.

### 3.2. Applicability

**Figure 2:**
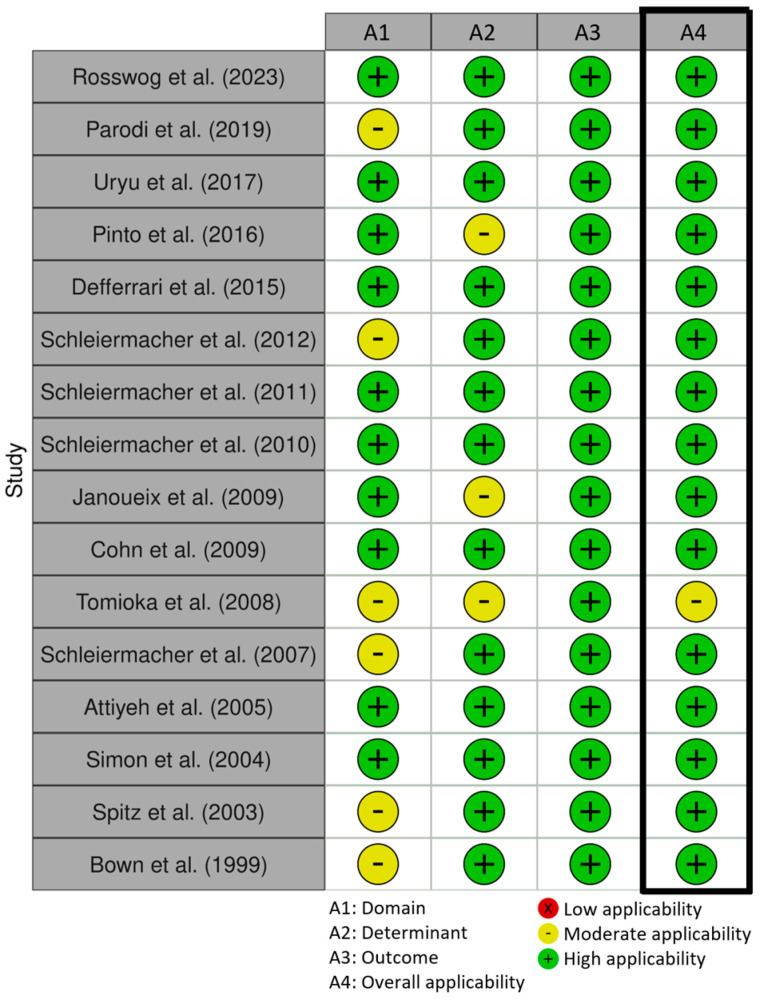
Applicability included studies.

The overall applicability was high for all included studies, except for Tomioka et al. (2019), which was rated as moderately applicable. Tomioka et al. (2019) investigated patients with a *MYCN*-non-amplified neuroblastoma, but did not stratify these tumors in risk groups. Furthermore, this study solely investigated genomic profiles and did not investigate single molecular alterations [35]. An overview of the applicability of all included studies can be found in Figure 2.

### 3.3. Risk of Bias

**Figure 3:**
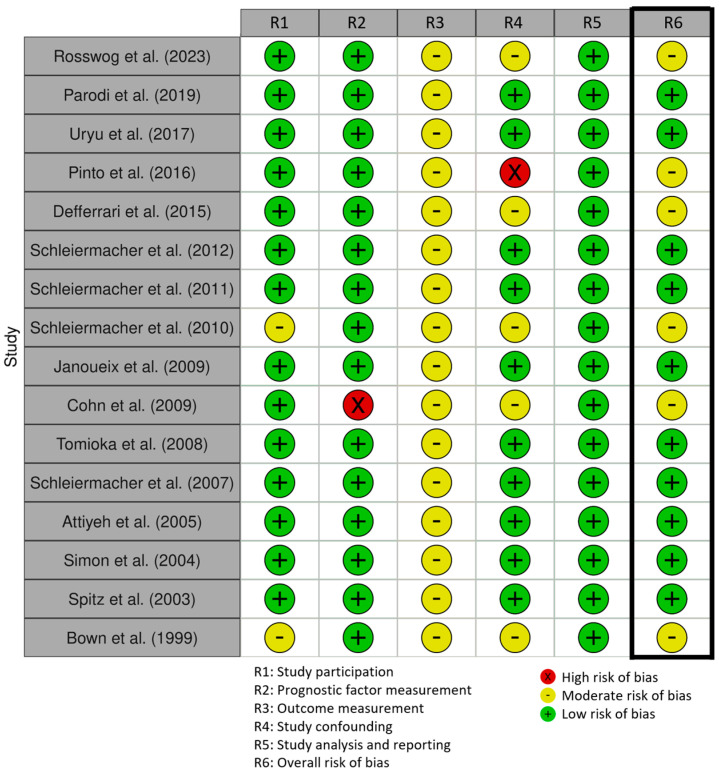
Risk of Bias included studies.

Ten studies were rated as ‘low RoB’, though they all scored ‘moderate RoB’ for ‘outcome measurement’, as they did not explain the definition and/or method used to estimate OS and EFS. The RoB of the other six studies was considered moderate, most of which scored moderate on either ‘study participation’ or ‘study confounding’ (Figure 3).

### 3.4. Findings

#### 3.4.1. Genomic Profile

Seven studies investigated the prognostic value of genomic profiles. All studies showed that patients with non-high-risk neuroblastoma with one or more SCAs were at higher risk for events (e.g., relapse or progression) and death compared to patients where no SCA was detected [24,25,27,30,31,32,35]. Four studies also estimated a Cox Proportional Hazard Model, and an SCA profile remained independently associated with both OS and EFS [25,30,31,32]. Data regarding genomic profile can be found in Table 2.

#### 3.4.2. Single Molecular Aberrations

Thirteen studies investigated the prognostic value of one or more single molecular aberrations in non-high-risk neuroblastomas (Table 3) [3,22,23,24,26,28,29,30,31,32,33,34,36]. Two molecular aberrations proved to be of prognostic value regarding both OS and EFS: 1p LOH and 17q gain. 1p LOH was investigated by two studies, both performing a univariate and multivariable analysis [22,36]. 1p LOH proved to be an independent prognostic factor for patients with a non-high-risk neuroblastoma in both univariate and multivariable analysis. All five studies concerning 17q gain reported a significant relation between the presence of this copy number variation (CNV) and both OS and EFS [23,24,30,31,32]. One Cox Proportional Hazard Model was estimated, which did not show 17q gain to be an independent prognostic factor. However, this study did not provide a HR, a 95% CI and/or *p*-value [30].

Seven studies investigated 1p deletion [3,24,30,31,32,33,34], and three studies investigated 2p gain [24,30,31]. Both CNVs proved to be associated with OS, but their effect on EFS varied in the different studies. However, there were two studies that did not find a significant relation between 1p deletion and OS, but did report a remarkably lower OS when 1p deletion was detected [30,32]. Two studies investigated 4p deletion, which showed an association with OS, but not with EFS [24,31]. The relation between 4p deletion and OS was only investigated by one study using the Kaplan Meier’s methodology [24]. Multiple studies investigated 3p deletion, which did not prove 3p deletion to be of prognostic value in non-high-risk neuroblastoma [24,30,31,33,34].

Furthermore, five molecular aberrations were only investigated by one study: 1q gain, 4p LOH, whole chromosome X deletion, chromosomal breakpoints and ALK mutation. Chromosomal breakpoints were correlated with worse outcome in OS and EFS [29] and 1q gain was related to inferior survival [24]. Whole chromosome X deletion was related to lower event rates [26]. ALK mutations [28] and 4p LOH [36] were not associated with outcome.

### 3.5. Quality of Evidence

We only included observational cohort studies, which provide high levels of confidence in prognostic studies. Therefore, the initial quality of evidence was rated as high. We downgraded the quality of evidence because of imprecision and the high probability of publication bias [37], after which we upgraded the quality of evidence because of moderate/large effects. We did not downgrade for RoB, inconsistency and indirectness. This resulted in an overall moderate quality of evidence. The GRADE criteria are further specified in Appendix E.

## 4. Discussion

This systematic review aimed to identify, evaluate and summarize the findings of all studies regarding the prognostic value of genetic aberrations in non-high-risk neuroblastoma not already included in the INRG risk classification system. This review has shown that genomic profile is a strong predictor of poor outcome in patients initially diagnosed with non-high-risk neuroblastoma. The presence of one or more SCA is strongly associated with both OS and EFS. Looking specifically into single molecular aberrations, 1p LOH, 1p deletion, 2p gain and 17q gain are associated with worse outcome in patients with a non-high-risk neuroblastoma, whereas no prognostic value of 3p deletion was found.

Recently, multiple risk stratification systems for other pediatric tumors have been revised by incorporating (more) SCAs. The Children’s Oncology Group (COG) has added 1p LOH and 16q LOH to the risk stratification for Wilms Tumor [38]. Furthermore, the COG has recently revised the INRG classification system by incorporating the presence of one or more SCAs as additional genomic biomarkers (specifically 1p LOH, 1q gain, 2p gain, 3p deletion, 4p deletion, 11q LOH and 17q gain) [11]. However, the ‘Société International Oncology Pediatrique’ (SIOP) uses the original INRG classification system, which only includes MYCN amplification and 11q aberration as biological markers [3]. This systematic review suggests, in accordance with COG criteria, that additional SCAs may be incorporated.

No articles regarding telomere maintenance mechanisms (TMMs) were identified in this review. Nevertheless, the recognition of the importance of TMMs in relation to outcome cannot be ignored [39,40,41,42,43,44]. Ackermann et al. (2018) identified 17 non-high-risk neuroblastoma patients with TMMs that showed similar outcomes to high-risk patients [39]. However, no studies regarding TMMs has been conducted specifically in non-high-risk neuroblastomas since children harboring TMMs are mainly high-risk patients [39,43].

This is the first systematic review that focusses on molecular aberrations in non-high-risk neuroblastoma. This is the most important strength of this study. Other systematic reviews have been conducted on this topic but did not focus on non-high-risk neuroblastoma [12,13,14,15]. The quality of evidence was rated moderate, which is the second highest possible result and is therefore considered a strength as well. However, this review also has limitations. Different methodologies have been used to assess SCAs. Studies using a targeted panel may have missed additional aberrations. Moreover, most of the studies only performed univariate analysis. The OS and/or EFS (with corresponding 95% CI or standard errors) were reported at variable time points (3, 4, 5 or 10 years). The intervals were often wide and standard errors large, which implies that the estimation of the OS/EFS is not accurate. Most studies did not report an HR/RR and the type of ratio used differed between studies. Furthermore, some studies performed a multivariable analysis, but only reported whether the association was significant and did not report an HR along with 95% confidence interval or *p*-value. Therefore, a meta-analysis could not be performed. We noticed that Defferrari et al. (2015) reported significant results regarding OS for almost all SCAs, whereas the findings regarding EFS were mostly insignificant. Moreover, the EFS of patients with a 1p deletion was higher than the OS of the patients in the same group, which is unusual. The number of articles included via snowballing is another limitation of this research, though an extensive database search was performed, and the search was thoroughly checked by a medical librarian. The articles that were included via snowballing all incorporated words in their title/abstract that were included in our search. Lastly, we have included studies that only distinguished between MYCN-amplified and MYCN-non-amplified tumors. Therefore, we could not entirely prevent that we included some high-risk patients: patients >18 months with stage 4 (or stage M) disease without MYCN amplification.

## 5. Conclusions

The findings of this systematic review indicate that SCAs are of paramount importance regarding the prognosis of patients with non-high-risk neuroblastoma. Based on our findings, we would recommend a risk-stratified clinical trial that investigates the possible improvement in outcome of neuroblastoma patients when incorporating 1p aberrations, 2p gain and 17q gain in the INRG classification. Furthermore, we would recommend a large multicenter study in which the prognostic value of genomic profile and of all individual CNVs is evaluated. This multicenter study should also investigate TMMs and prognostic value of molecular aberrations that have not been investigated in non-high-risk neuroblastoma yet, or have been investigated by one study only (1q gain, 4p LOH, 4p deletion and number of chromosomal breakpoints). We believe that we can improve the current INRG classification system as well as the therapeutic approach to these seemingly non-high-risk neuroblastoma patients, thereby reducing the number of patients with a neuroblastoma that succumb to their disease.

## Figures and Tables

**Table 1:** Study characteristics.

Author (Year)	Year	Centre/Country	Participants	Prognostic Factor	Outcome	Median Follow-Up in Months (Range)
Rosswog et al. [28]	2023	Germany	365 LR/IR NBL patients	ALK mutations	EFS, OS	Cohort 1: 84 * Cohort 2: NR Cohort 3: NR
Parodi et al. [26]	2019	23 centers in Italy	174 NCA profile, non-MYCN amplified NBL patients	Whole chr. X deletion	EFS	(0–164)
Uryu et al. [36]	2017	Tokyo University hospital and many other Japanese hospitals	97 IR NBL patients	1p LOH, 4p LOH	OS	NR
Pinto et al. [27]	2016	Five children’s hospitals in USA: Chicago (2), Toronto, Philadelphia, Minnesota	105 LR/IR NBL patients	All possible CNV	EFS, OS	NR
Defferrari et al. [24]	2015	Austria, Belgium, Czech Republic, France, Italy, Norway, Portugal, Spain, Sweden, United Kingdom	98 NBL patients aged >12 months without MYCN amplification	CNVs chr.1, 2, 3, 4, 7, 9, 11, 12, 14 and 17	EFS, OS	NR
Schleiermacher et al. [32]	2012	Australia, Germany, Italy, Japan, North America, Spain	505 non-MYCN amplified NBL patients	CNVs 1p, 11q, 17q	EFS, OS	63 (0–167)
Schleiermacher et al. [31]	2011	Austria, Belgium, France, Italy, Norway, Portugal, Spain, Sweden United Kingdom	218 non-MYCN amplified NBL patients aged <12 months	All possible CNV	PFS	60*
Schleiermacher et al. [29]	2010	Institut Curie (Paris, France)	145 non-MYCN amplified NBL patients	Chromosomal breakpoints	PFS, OS	49 (0–229) *
Cohn et al. [3]	2009	Australia, Germany, Italy, Japan, North America, Spain	495 LR NBL	1p deletion	EFS, OS	62 *
Janoueix et al. [25]	2009	France and other (not specified) European countries	286 LR/IR NBL patients	All possible CNVs	PFS	Institut Curie: 46 (3–183) * Additional patients: 58 (0–229) *
Tomioka et al. [35]	2008	Various institutions in Japan	76 non-MYCN amplified NBL patients	All possible CNVs	OS	NR
Schleiermacher et al. [30]	2007	Centres of the Société Française des Cancers de l’Enfant	139 non-MYCN amplified NBL patients	CNVs chromosome 1, 2, 3, 11 and 17	EFS, OS	57.3 (17–194)
Attiyeh et al. [22]	2005	NR	744 non-MYCN amplified and 524 LR/IR NBL patients	1p36 LOH	EFS, OS	36 *
Simon et al. [33]	2004	Germany	908 LR/IR NBL patients	1p deletion, 3p deletion	EFS, OS	50 (0–160)
Spitz et al. [34]	2003	50 children’s hospitals (German multicenter trial)	145 non-MYCN amplified NBL patients	1p deletion, 3p deletion	EFS	NR
Bown et al. [23]	1999	6 European centers	210 non-MYCN amplified NBL patients	17q gain	OS	30 *

* Median was derived from eligible patients (including high-risk patients) instead of included patients (solely LR/IR or non-MYCN amplified). Abbreviations: CNV, copy number variations; EFS, event-free survival; IR, intermediate risk; LR, low risk; NBL, neuroblastoma; NCA, numerical chromosomal alterations, NR, not reported; OS, overall survival; PFS, progression-free survival.

**Table 2:** Prognostic value of genomic profile: NCA versus SCA.

Author (Year)	OS (%)	RR/HR	95% CI/S.E.	*p*-Value	EFS/PFS (%)	RR/HR	95% CI/S.E.	*p*-Value
Pinto et al. (2016) [27] *Univariate analysis*	NCA: 100 SCA: 88.1	13.7	NA 95% CI 0.78–240	0.07	NCA: 91 SCA: 68		S.E. 3.6 S.E. 8.3	0.0083
Defferrari et al. (2015) [24] *Univariate analysis*	12-18 months No SCA: 100 SCA: 100 >18 months No SCA: 100 SCA: 66.8		NA NA 95% CI 47.4–80.5	NA 0.003	12-18 months No SCA: 100 SCA: 95.5 >18 months No SCA: 75.0 SCA: 46.1		NA 95% CI 49.1–89.0 95% CI 29.6–61.0	0.45 0.023
Schleiermacher et al. (2012) [32] *Univariate analysis*	NCA: 88 SCA: 71		S.E. 3.2 S.E. 2.5	<0.001	NCA: 79 SCA: 53		S.E. 3.9 S.E. 2.7	<0.001
Schleiermacher et al. (2012) [32] *Multivariable analysis*		1.8	NR	0.05		1.7	NR	0.01
Schleiermacher et al. (2011) [31] *Univariate analysis*					NCA: 92.0 SCA: 70.7 Silent: 62.5		S.E. 2.1 S.E. 6.6 S.E. 17.1	<0.001
Schleiermacher et al. (2011) [31] *Multivariable analysis*						5.24	95% CI 2.4–11.4	<0.001
Janoueix et al. (2009) [25] *Univariate analysis*					LR NCA: 93.0 LR SCA: 80.0 IR NCA: 93.7 IR SCA: 73.0		S.E. 2.8 S.E. 7.3 S.E. 2.3 S.E. 6.5	0.006 <0.001
Janoueix et al. (2009) [25] *Multivariable analysis*						4.5	95% CI 2.4-8.4	<0.001
Tomioka et al. (2008) [35] *Univariate analysis*		3.41	95% CI 1.32-8.82	0.010				
Schleiermacher et al. (2007) [30] *Univariate analysis*	NCA: 98.6 SCA: 75.8		S.E. 1.4 S.E. 5.5	<0.001	NCA: 88.6 SCA: 43.5		S.E. 3.8 S.E. 6.3	<0.001
Schleiermacher et al. (2007) [30] *Multivariable analysis*						4.62	95% CI 2.03–10.5	<0.001

Comparison of OS and EFS (Pinto et al., 2016; Defferari et al., 2015; Schleiermacher et al., 2007)/PFS (Schleiermacher et al., 2012; Schleiermacher et al., 2011, Janoueix et al., 2009) of children with a NCA or SCA genetic profile tumor. Some articles provided a risk ratio (Schleiermacher et al., 2012; Schleiermacher et al., 2007) or a hazard ratio (Pinto et al., 2016; Schleiermacher et al., 2011; Janoueix et al., 2009; Tomioka et al., 2008). NCA profile was the reference category in these articles. The follow-up period for the outcome measurements is either 4 years (Schleiermacher et al., 2012; Janoueix et al., 2009; Schleiermacher et al., 2007) or 5 years (Defferari et al., 2015; Scheiermacher et al., 2011; Tomioka et al., 2008). Pinto et al. (2016) had a follow-up period of 10 years for OS and 5 years for EFS. Abbreviations: CI, confidence interval; EFS, event-free survival; HR, hazard ratio; IR, intermediate risk; LR, low risk; NCA, numerical chromosomal aberrations; NR, not reported; NS, not significant; OS, overall survival; PFS, progression-free survival; RR, risk ratio; SCA, segmental chromosomal aberrations; S.E., standard error.

**Table 3:** Prognostic value of single molecular aberrations.

Genetic Alteration	Author (Year)	OS (%)	HR/RR	95% CI/S.E.	*p*-Value	EFS/PFS (%)	HR/RR	95% CI/S.E.	*p*-Value
1p LOH	Uryu et al. (2017) [36] *Univariate analysis* Uryu et al. (2017) [36] *Multivariable analysis*	No: 91 * Yes: 31 *	3.87	NR 95% CI 1.0–14.3	0.0014 0.051				
	Attiyeh et al. (2005) [22] *Univariate analysis*	No: 87 Yes: 83		S.E. 2 S.E. 5	0.05	No: 79 Yes: 62		S.E. 2 S.E. 6	<0.001
	Attiyeh et al. (2005) [22] *Multivariable analysis*						2.92		0.002
1p deletion	Defferrari et al. (2015) [24] *Univariate analysis*	12–18 months No: 92.7 Yes: 70.0 >18 months No: 88.3 Yes: 47.4		95% CI 83.2–96.9 95% CI 41.5–86.5 95% CI 74.1–95.0 95% CI 14.1–75.7	0.014 0.004	12–18 months No: 73.6 Yes: 72.2 >18 months No: 59.2 Yes: 54.6		95% CI 61.7–82.4 95% CI 45.6–87.4 95% CI 43.1–72.2 95% CI 22.9–78.0	0.904 0.487
	Schleiermacher et al. (2012) [32] *Univariate analysis*	No: 79 Yes: 72		S.E. 2.6 S.E. 3.3	0.11	No: 63 Yes: 55		S.E. 2.9 S.E. 3.7	0.06
	Schleiermacher et al. (2011) [31] *Univariate analysis*					No: 88.5 Yes: 74.3		S.E. 2.3 S.E. 9.9	0.09
	Cohn et al. (2009) [3] *Univariate analysis*	No: 99 Yes: 100		S.E. 1.0 NA	NR	No: 94 Yes: 78		S.E. 2.0 S.E. 10.0	NR
	Schleiermacher et al. (2007) [30] *Univariate analysis*	No: 87.9 Yes: 80.0		S.E. 3.2 S.E. 10.3	NS	No: 67.0 Yes: 59.3		S.E. 4.4 S.E. 12.9	NS
	Schleiermacher et al. (2007) [30] *Multivariable analysis*								NS
	Simon et al. (2004) [33] *Univariate analysis*	No: 97.3 Yes: 85.7		S.E. 1.2 S.E. 9.4	0.027	No: 84.2 Yes: 49.6		S.E. 2.6 S.E. 14.2	<0.001
	Simon et al. (2004) [33] *Multivariable analysis*		3.0	95% CI 1.3–7.0	0.009		3.6	95% CI 1.5–8.7	0.005
	Spitz et al. (2003) [34] *Univariate analysis*					No: 71 Yes: 44		S.E. 6 S.E. 23	0.02
1q gain	Defferrari et al. (2015) [24] *Univariate analysis*	12–18 months No: 90.7 Yes: 70.0 >18 months No: 84.3 Yes: 40.0		95% CI 81.1–95.5 95% CI 11.1–80.4 95% CI 69.3–92.4 95% CI 5.2–75.3	0.001 0.005	12–18 months No: 75.0 Yes: 50.0 >18 months No: 59.9 Yes: 40.0		95% CI 64.1–83.0 95% CI 11.1–80.4 95% CI 44.6–72.1 95% CI 5.2–75.3	0.192 0.389
2p gain	Defferrari et al. (2015) [24] *Univariate analysis*	12–18 months No: 90.8 Yes: 70.6 >18 months No: 84.5 Yes: 6.5		95% CI 80.3–95.8 95% CI 43.2–86.6 95% CI 68.1–92.9 95% CI 30.8–81.8	0.014 0.027	12–18 months No: 76.6 Yes: 52.9 >18 months No: 62.2 Yes: 38.5		95% CI 65.2–84.6 95% CI 27.6–73.0 95% CI 46.1–74.7 95% CI 14.1–62.8	0.064 0.089
	Schleiermacher et al. (2011) [31] *Univariate analysis*					No: 89.4 Yes: 66.3		S.E. 2.2 S.E. 10.4	0.002
	Schleiermacher (2007) [30] *Univariate analysis*	No: 90.3 Yes: 68.6		S.E. 2.9 S.E. 10.7	<0.001	No: 72.1 Yes: 33.1		S.E. 4.3 S.E. 10.9	<0.001
	Schleiermacher et al. (2007) [30] *Multivariable analysis*								NS
3p deletion	Defferrari et al. (2015) [24] *Univariate analysis*	12–18 months No: 87.9 Yes: 83.3 >18 months No: 79.4 Yes: 83.3		95% CI 78.5–93.3 95% CI 27.3–97.5 95% CI 64.9–88.4 95% CI 27.3–97.5	0.301 0.811	12–18 months No: 75.6 Yes: 42.9 >18 months No: 59.9 Yes: 42.9		95% CI 64.9–93.3 95% CI 9.8–73.4 95% CI 44.6–72.1 95% CI 9.8–73.4	0.083 0.427
	Schleiermacher et al. (2011) [31] *Univariate analysis*					No: 86.6 Yes: 100		S.E. 2.4 S.E. 4.3	NS
	Schleiermacher et al. (2007) [30] *Univariate analysis*	No: 92.1 Yes: 64.0		S.E. 2.7 S.E. 10.4	<0.001	No: 72.5 Yes: 36.5		S.E. 4.4 S.E. 10.0	<0.001
	Schleiermacher et al. (2007) [30] *Multivariable analysis*								NS
	Simon et al. (2004) [33] *Univariate analysis*	No: 96.1 Yes: 83.3		S.E. 1.7 S.E. 15.2	0.268	No: 83.7 Yes: 0		S.E. 3.3 NA	<0.001
	Simon et al. (2004) [33] *Multivariable analysis*		NR	NR	0.491		NR	NR	0.814
	Spitz et al. (2003) [34] *Univariate analysis*					No: 82 Yes: 0		S.E. 6 NA	0.001
4p LOH	Uryu et al. (2017) [36] *Univariate analysis* Uryu et al. (2017) [36] *Multivariable analysis*	No: 87 * Yes: 66 *	2.49	NR 95% CI 0.5–10.1	0.04 0.25				
4p deletion	Defferrari et al. (2015) [24] *Univariate analysis*	12–18 months No: 89.1 Yes: 50.0 >18 months No: 81.6 Yes: 50.0		95% CI 79.8–94.2 95% CI 5.8–84.5 95% CI 67.1–90.2 95% CI 5.8–84.5	<0.001 0.016	12–18 months No: 75.5 Yes: 25.0 >18 months No: 60.3 Yes: 25.0		95% CI 65.1–83.2 95% CI 0.9–66.5 95% CI 45.6–72.2 95% CI 0.9–66.5	0.042 0.260
	Schleiermacher et al. (2011) [31] *Univariate analysis*					No: 87.7 Yes: 78.6		S.E. 2.3 S.E. 11.0	NS
17q gain	Defferrari et al. (2015) [24] *Univariate analysis*	12–18 months No: 94.0 Yes: 74.5 >18 months No: 89.2 Yes: 65.4		95% CI 81.6–98.1 95% CI 55.2–86.5 95% CI 68.9–96.5 95% CI 41.8–81.3	0.002 0.009	12–18 months No: 78.3 Yes: 60.9 >18 months No: 62.8 Yes: 48.5		95% CI 65.1–86.9 95% CI 42.4–75.1 95% CI 43.2–77.3 95% CI 28.2–66.1	0.050 0.211
	Schleiermacher et al. (2012) [32] *Univariate analyis*	No: 86 Yes:72		S.E. 2.9 S.E. 4.3	<0.001	No: 75 Yes: 49		S.E. 3.6 S.E. 4.7	<0.001
	Schleiermacher et al. (2011) [31] *Univariate analyis*					No: 91.2 Yes: 69.0		S.E. 2.4 S.E. 7.4	<0.001
	Schleiermacher et al. (2007) [30] *Univariate analysis*	No: 97.2 No: 75.9		S.E. 2.0 S.E. 5.7	<0.001	No: 85.0 Yes: 44.7		S.E. 4.2 S.E. 6.5	<0.001
	Schleiermacher et al. (2007) [30] *Multivariable analysis*								NS
	Bown et al. (1999) [23] *Univariate analysis*	No: 90 Yes: 38		95% CI 81–95 95% CI 21–54	<0.001				
X-deletion	Parodi et al. (2019) [26] *Univariate analysis*					No: 83.1 Yes: 100		95% CI 73.0–89.7 NA	0.002
Chromosomal breakpoints	Schleiermacher et al. (2010) [29] *Univariate analyis*	1–3: 92 * 4–6: 53 * >7: 28 *		NR	<0.001	1–3: 65 * 4–6: 30 * >7: 28 *		NR	<0.001
ALK mutation	Rosswog et al. (2023) [28] *Univariate analysis*	No: 96 Yes: 92		NR	0.480	No: 78 Yes: 70		NR	0.330

Comparison of OS and EFS (Rosswog et al., 2023; Parodi et al., 2019; Defferari et al., 2015; Cohn et al., 2009; Schleiermacher et al., 2007, Attiyeh et al., 2005, Simon et al., 2004, Spitz et al., 2003)/PFS (Schleiermacher et al., 2012; Schleiermacher et al., 2011, Schleiermacher et al., 2010) of children with a NBL with and without 1p LOH, 1p deletion, 1q gain, 2p gain, 3p deletion, 4p deletion, 4p LOH, 17 gain, whole chromosome X deletion, chromosomal breakpoints and ALK mutation. Some articles provided a risk ratio (Uryu et al., 2017) or a hazard ratio (Attiyeh et al., 2005; Simon et al., 2004). NCA profile was the reference category in these articles. The follow-up period for the outcome measurements is either 3 years (Attiyeh et al., 2007; Simon et al., 2004; Spitz et al., 2003), 4 years (Schleiermacher et al., 2012; Schleiermacher et al., 2007) or 5 years (Rosswog et al., 2023; Parodi et al., 2019; Defferari et al., 2015; Schleiermacher et al., 2011; Cohn et al., 2009; Bown et al., 1999). Uryu et al. (2017) and Schleiermacher et al. (2010) reported OS/PFS/RR till last follow up. Abbreviations: CI, confidence interval; EFS, event-free survival; HR, hazard ratio; LOH, loss of heterozygosity; NA, not applicable; NR, not reported; NS, not significant; OS, overall survival; PFS, progression-free survival; RR, risk ratio; S.E., standard error. * Percentage derived from figure in original article (no exact data available).

## Data Availability

No new data were created or analyzed in this study. Data sharing is not applicable to this article.

## Supplementary Materials

The following supporting information can be downloaded at: https://www.mdpi.com/article/10.3390/cancers17010013/s1.

## Appendix A. PICOTS Framework

- Patient population: pediatric patients (<18 years old) withLR/IR NBL.
- Intervention: the presence of molecular aberrations.
- Comparator: independent of molecular aberrations that are already included in the risk stratification: MYCN amplification and 11q aberration.
- Outcome: OS and EFS.
- Timing: molecular marker is measured at diagnosis.
- Setting: secondary and tertiary healthcare centers.

Research question: which molecular aberrations (not incorporated in the current risk stratification) are associated with OS and EFS in children (<18 years) initially diagnosed with a non-high-risk neuroblastoma?

## Appendix B. Database Search

### Appendix B.1. PubMed

#1 Pediatrics
“child” [Mesh] OR “pediatrics” [Mesh] OR “adolescent” [Mesh] OR “infant” [Mesh] OR “child*” [Title/Abstract] OR “pediat*” [Title/Abstract] OR “paediat*” [Title/Abstract] OR “neonat*” [Title/Abstract] OR “kids” [Title/Abstract] OR “kid” [Title/Abstract] OR “teen*” [Title/Abstract] OR “adolesc*” [Title/Abstract] OR “newborn*” [Title/Abstract] OR “infan*” [Title/Abstract]
#2 Neuroblastoma
“neuroblastoma” [Mesh] OR “neuroblast*” [Title/Abstract] OR “ganglioneuroma*” [Title/Abstract] OR “ganglioneuroblastoma*” [Title/Abstract]
#3 Low/intermediate risk
“low-risk” [Title/Abstract] OR “intermediate-risk” [Title/Abstract] OR “low risk” [Title/Abstract] OR “intermediate risk” [Title/Abstract]
#4 Neuroblastoma
“mutation” [Mesh] OR “mutat*” [Title/Abstract] OR “aberrat*” [Title/Abstract] OR “prognostic marker*” [Title/Abstract] OR “prognostic factor*” [Title/Abstract] OR “tumor marker*” [Title/Abstract] OR “tumour marker*” [Title/Abstract] OR “molecular marker*” [Title/Abstract] OR “amplificat*” [Title/Abstract] OR “delet*” [Title/Abstract] OR “gain*” [Title/Abstract] OR “alterat*” [Title/Abstract] OR “NCA*” [Title/Abstract] OR “SCA*” [Title/Abstract]
#5 Event
“Death” [Mesh] OR “recurrence” [Mesh] OR “disease progression” [Mesh] OR “hospitalization” [Mesh] OR “mortality” [Mesh] OR “survival” [Mesh] OR “Death*” [Title/Abstract] OR “recur*” [Title/Abstract] OR “progress*” [Title/Abstract] OR “hospitali*” [Title/Abstract] OR “mortalit*” [Title/Abstract] OR “surviv*” [Title/Abstract] OR “event*” [Title/Abstract] OR “deceas*” [Title/Abstract] OR “relaps*” [Title/Abstract]

#1 AND #2 AND #3 AND #4 AND #5

### Appendix B.2. Embase

(‘child’/exp OR ’pediatric’/exp OR ’adolescent’/exp OR ’juvenile’/exp OR ‘infant’/exp OR ’child*’:ti,ab,kw OR ‘pediat*’:ti,ab,kw OR ’paediat*’:ti,ab,kw OR ’neonat*’:ti,ab,kw OR ’kids’:ti,ab,kw OR ‘kid’:ti,ab,kw OR ’teen*’:ti,ab,kw OR ’adolesc*’:ti,ab,kw OR ‘newborn*’:ti,ab,kw OR ’infan*’:ti,ab,kw) AND (‘neuroblastoma’/exp OR ‘neuroblast*’:ti,ab,kw OR ‘ganglioneuroma*’:ti,ab,kw OR ‘ganglioneuroblastoma*’:ti,ab,kw) AND (‘mutation’/exp OR ’tumor marker’/exp OR ‘mutat*’:ti,ab,kw OR ’aberrat*’:ti,ab,kw OR ‘prognostic marker*’:ti,ab,kw OR ’prognostic factor*’:ti,ab,kw OR ’tumor marker*’:ti,ab,kw OR ’tumour marker*’:ti,ab,kw OR ’molecular marker*’:ti,ab,kw OR ’amplificat*’:ti,ab,kw OR ’delet*’:ti,ab,kw OR ’gain*’:ti,ab,kw OR ’alterat*’:ti,ab,kw OR ’NCA*’:ti,ab,kw OR ’SCA*’:ti,ab,kw) AND (’low risk population’/exp OR ’low-risk’:ti,ab,kw OR ’intermediate-risk’:ti,ab,kw OR ‘low risk’:ti,ab,kw OR ’intermediate risk’:ti,ab,kw) AND (’death’/exp OR ’recurrent disease’/exp OR ’hospitalization’/exp OR ’mortality’/exp OR ’survival’/exp OR ‘death*’:ti,ab,kw OR ’recur*’:ti,ab,kw OR ‘progress*’:ti,ab,kw OR ‘hospitali*’:ti,ab,kw OR ‘mortalit*’:ti,ab,kw OR ’surviv*’:ti,ab,kw OR ’event*’:ti,ab,kw OR ‘deceas*’:ti,ab,kw OR ‘relaps*’:ti,ab,kw)

### Appendix B.3. Cochrane

#1 Pediatrics
(child* OR pediat* OR paediat* OR neonat* OR kids OR kid OR teen* OR adolesc* OR newborn* OR infan*):ti,ab,kw
#2 Neuroblastoma
(neuroblast* OR ganglioneuroma* OR ganglioneuroblastoma*):ti,ab,kw
#3 Low/intermediate risk
(“low-risk” OR “intermediate-risk” OR “low risk” OR “intermediate risk”):ti,ab,kw
#4 Neuroblastoma
(mutat* OR aberrat* OR “prognostic” NEXT marker* OR “prognostic” NEXT factor* OR “tumor” NEXT marker* OR “tumour” NEXT marker* OR “molecular” NEXT marker* OR amplificat* OR delet* OR gain* OR NCA* OR SCA* OR alterat*):ti,ab,kw
#5 Event
(death* OR recur* OR progress* OR hospitali* OR mortalit* OR surviv* OR event* OR deceas* OR relaps*):ti,ab,kw

#1 AND #2 AND #3 AND #4 AND #5

### Appendix B.4. Cochrane

children|pediatric|infant neuroblastoma|ganglioneuroma|ganglioneuroblastoma low-risk|intermediate-risk
mutation|aberration|alteration|amplification|deletion|gain|NCA|SCA|prognostic death|recurrence|progression|mortality|survival|event|relapse\

## Appendix C. Applicability

### Appendix C.1. Scoring Applicability

High applicability:

- All three criteria were rated ‘high applicability’.
- One criterion was rated ‘moderate applicable’.

Moderate applicability:

- Two or more criteria were rated ‘moderate applicability’.
- One criterion was rated ‘low applicability’.

Low applicability:

- Two or more criteria were rated ‘low applicability’.

### Appendix C.2. Applicability Tool

Representativeness of domain:

- High applicability = pediatric patients (<18 years old) diagnosed with a LR/IR neuroblastoma using the current risk stratification or when the tumor would have been classified as non-high-risk neuroblastoma when using the current risk stratification.
- Moderate applicability = (pediatric) patients diagnosed with a MYCN-non-amplified neuroblastoma:
  ○ *MYCN*-non-amplified neuroblastomas frequently classify as non-high-risk. However, metastasized non-*MYCN*-amplified neuroblastomas in patients older than 18 months do classify as high risk. The same applied to metastasized non-*MYCN*-amplified neuroblastomas in patients younger than 18 months with an 11q aberration [3].

- Low applicability = none of the above.

Representativeness of determinant:

- High applicability = single molecular alteration(s) or mutation(s) were investigated.
- Moderate applicability = genomic profile types and/or number of chromosomal breakpoints were investigated, but single molecular alteration or mutation(s) were not investigated.
- Low applicability = none of the above.

Representativeness of outcome:

- High applicability = OS and/or EFS (PFS was also accepted) were reported.
- Low applicability = none of the above.

## Appendix D. Risk of Bias

### Appendix D.1. Scoring RoB

Low RoB:

- All domains were rated ‘low RoB’.
- Only ‘outcome measurement’ was rated ‘moderate RoB’.

Moderate RoB:

- No domain was rated ‘high RoB’ + two or more domains were rated ‘moderate RoB’.
- One domain was rated ‘high RoB’ + one or two domains were rated ‘moderate RoB’.

High RoB:

- Two or more domains were rated ‘high RoB’.
- ‘Study confounding’ was rated ‘high RoB’ and two or more domains were rated ‘moderate RoB’.
- One domain was rated ‘high RoB’ and three or more domains were rated ‘moderate RoB’.

### Appendix D.2. Specifications QUIPS Tool for RoB

**Table A1:** Specifications QUIPS tool for RoB.

Domain in QUIPS	More (+) or Less (−) Determinative for Overall RoB		Specifications/Removal of Subdomains
Study participation	+	Source of target population and in- and exclusion criteria might relate to OS and/or EFS.	Source of target population: key characteristics had to include pediatric patients with a neuroblastic tumor (LR/IR). Baseline characteristics: key characteristics had to include patients with a neuroblastic tumor (LR/IR).
Study attrition	−	Domain removed *	Not applicable
Prognostic factor measurement	+	Correct and uniform measurement of the prognostic factor is crucial in determining whether a prognostic factor is valid.	Valid and reliable measurement of PF: detailed description how molecular aberrations/genetic profiles were measured.
Outcome measurement	−	Detailed method description is less relevant as our outcome measures were easily objectifiable and are therefore less prone to bias.	Valid and reliable measurement of outcome: description on method of the outcome measurements (OS/EFS).
Study confounding	++	We sought to investigate the independent prognostic value of molecular aberrations, thus potential confounders might severely alter our results.	Studies had to be adjusted for known confounders (MYCN amplification and 11q aberration).
Statistical analysis and reporting	+	Appropriate statistical analysis and reporting were used to calculate outcomes measures.	Reporting of results: predetermined outcome measurements were reported, preferably sourced from a study protocol. Selected statistical model: outcome measurements were appropriate for our research question (i.e., OS, EFS and PFS). Strategy for model building: model building was not indicated for our research question, thus this subdomain was removed.

* This domain judges RoB based on the likelihood that the relationship of the prognostic factor or outcome measurements are different for completing and non-completing participant. We solely identified retrospective cohort prognostic studies. As these studies only include patients in which both determinant and outcome are known, we could not identify completing/non-completing participants. In addition, we did not expect this domain to have a large effect on overall RoB.

## Appendix E. GRADE Criteria

Grading down:

1. RoB: no downgrade
  - Ten studies had an overall low RoB, and six had an overall moderate RoB, which was predominantly because they did not perform a multivariable analysis to adjust for known confounders.
2. Imprecision: downgrade
  - Multiple studies reported wide confidence intervals and high standard deviations.
3. Inconsistency: no downgrade
  - Most results regarding single molecular aberrations and the findings regarding genomic profile were consistent. Only the studies investigating 1p deletion and 3p deletion showed conflicting results regarding both OS and EFS/PFS.
4. Indirectness: no downgrade
  - Fifteen studies were highly applicable to our research question. One study solely investigated prognostic value of genomic profile in MYCN-non-amplified tumors (instead of non-high-risk neuroblastomas).
5. Publication bias: downgrade
  - We found no indication of publication bias since there were studies showing conflicting results and non-significant results were published as well (also in smaller studies). However, the risk of publication bias is larger in observational studies than for randomized controlled trials. Furthermore, since preregistration is not required for observational studies, we were not able to address publication bias properly.

Grading up:

1. Moderate/large effect: upgrade
  - It is difficult to determine the effect size based on OS and EFS/PFS. Therefore, we looked at studies that reported a RR or HR (n = 11). Eight of them reported an effect size larger than three and four of these reported ratios larger than 4.5.
2. Dose-response gradient: no upgrade
  - Not applicable to this study.
3. All plausible confounders decrease an apparent treatment effect: no upgrade
  - Not applicable in prognostic studies.
